# The Utility of Pan-Scan Imaging in Older Patients After Ground-Level Falls: A Comparative Study

**DOI:** 10.7759/cureus.98325

**Published:** 2025-12-02

**Authors:** Damian Hernandez, Stacey L Tannenbaum, Brooke Davis, Allison Draper, Alexander A Fokin, Mario Gomez

**Affiliations:** 1 Department of General Surgery, Broward Health Medical Center, Fort Lauderdale, USA; 2 Surgery, Florida Atlantic University Charles E. Schmidt College of Medicine, Boca Raton, USA; 3 Trauma and Acute Care Surgery, Delray Medical Center, Delray Beach, USA; 4 Surgery, Florida International University, Herbert Wertheim College of Medicine, Miami, USA

**Keywords:** ground level fall, ground-level fall, low-energy trauma, low intensity blunt trauma, pan-scan, selective ct, trauma

## Abstract

Background: This study assessed pan-scan versus selective CT (SCT) for ground-level falls in older adults.

Methods: This was a retrospective cohort study of adults aged 65+ who sustained a ground-level fall. Patients underwent SCT (brain, cervical spine, plus one additional CT scan) or pan-scan (brain, cervical/thoracic/lumbar spine, abdomen/pelvis, and chest).

Results: Two hundred three patients received SCT (n=105, 51.7%) or pan-scan (n=98, 48.3%). Significantly more consultations based on incidental findings were found in the pan-scan group (P=0.020). Significantly more operative interventions were seen among those who had a pan-scan (n=43, 43.9%) compared with those who had SCT (n=19, 18.1%; p<0.001). Patients who received SCT had a significantly shorter hospital course (average 4.0 days vs 6.0 days; p=0.001), without mortality difference (p=0.168). Multivariable linear regression models predicted that having a pan-scan increased hospital length of stay (R2=0.603).

Conclusions: Use of a physical exam as a guide for diagnostic imaging reduces unnecessary imaging without compromising patient outcomes and may reduce hospital length of stay.

## Introduction

People are living longer than ever before, and the population is continually mushrooming with older individuals aged 65 plus. The fifth leading cause of death for older adults is trauma; trauma accounts for one in four admissions to a trauma service in the US [1]. Ground-level falls (GLF) are the most common mechanism of blunt trauma for patients 65 years and older presenting to a trauma center [2] and are the source of an increased risk of mortality, especially for those 70 and older [3]. According to the US Department of Health and Human Services' Healthy People 2030, a federal initiative that looks at national data regarding specific objectives, falls in older adults accounted for 78 deaths per 100,000 people in 2021; the goal of Healthy People 2030 is to lower this to 64.4 deaths per 100,000 people [4]. The diagnosis of older patients presenting with a GLF is enhanced when using appropriate imaging and diagnostic tests, including computed tomography (CT), focused assessment with sonography in trauma, and pan-scan - also called whole-body scan, as per Advance Trauma Life Support protocol. Recently an algorithm intended to discern whether to give older adults a pan-scan or selective CT (SCT) after a bunt trauma from any injury was created; however, it was not specific to GLFs [5]. Thus, little is known about the utility and benefits of SCT versus pan-scan imaging in older adult patients who experienced a GLF.

Opinions in the literature are mixed regarding the utilization of pan-scans in older patients who had a GLF [6-8]. Older patients sustain more severe injuries even in lower energy mechanisms when compared to their younger counterparts but no guidelines are currently in place that fully address the differences in this at-risk population of older individuals. Studies show that incidental findings on CT are more common.

In older adults, however, not all have clinical significance in the trauma setting [7,9,10]. Incidental findings can increase hospital length of stay without a decrease in mortality [6,7,11,12]. Radiology groups have put forth guidelines for imaging in trauma patients that have been found to significantly reduce cost and radiation exposure, but these guidelines do not stratify patients by age or make specific recommendations for this older population group [13]. Thus, the purpose of this study is to evaluate adult patients 65 years and older who experienced a GLF for the utility of pan-scan imaging compared with selective imaging techniques based on changes in treatment approaches or outcomes. We hypothesize that selective CT scans will be as discerning of injury discovery as pan-scans without compromising clinical outcomes.

## Materials and methods

Study sample and procedures

This was a retrospective cohort study of data collected on patients 65 years of age and older who sustained a GLF and were admitted at a local urban level 2 trauma center. All procedures followed were in accordance with the ethical standards of the committee on human experimentation of the hospital system’s Institutional Review Board and given an exempt status. The study did not require informed consent because it was a retrospective study reporting on de-identified data. Patients were selected for this study if they were evaluated by the trauma team. Patient selection was also based on ICD-10 codes specific to GLFs discovered by querying the electronic medical records and extracting relevant variables within the timeframe of January 1, 2021, to March 30, 2022. Older adults were defined as patients aged 65 and older [14]. Inclusion criteria were all older adult patients evaluated by the trauma team who presented with a GLF and received any CT imaging. Patients were excluded if they left the hospital against medical advice, had no CT performed, or were discharged directly from the trauma bay or emergency department.

Demographic data were extracted from the electronic medical record. Also retrieved were Glasgow Coma Scale (GCS), vital signs on presentation (such as pulse rate, respiration rate, and blood pressure), chief complaint on presentation, home medications including use of antiplatelet/anticoagulation agents, comorbidities including underlying dementia, blood alcohol level or documentation of intoxication - blood alcohol above the legal limit of 0.08 and, presenting blood levels of hemoglobin (mg/dL), international normalised ratio (INR), and albumin (g/dL). Injury characteristics collected were the specific mechanism of fall, injury severity score (0-75), and specific injuries as identified on CT imaging. Imaging characteristics retrieved from the electronic medical record included the timing of imaging (time from entering trauma bay or emergency department to the scanning images). For this study, the term congruency is defined as whether the imaging findings were aligned with the patient’s chief complaint.

Patients were designated as either having: (1) selective imaging, defined as CT of the brain without contrast, CT of the cervical spine without contrast, and at least one additional CT scan with or without contrast related to the chief complaint of the patient at presentation, or (2) pan-scan, defined as a CT of the five body regions with contrast enhancement for chest and abdomen. Negative CT is defined as imaging in which there were no acute injuries or subacute injuries requiring action and/or monitoring. Imaging characteristics were obtained from radiology reports and not from actual images. Focused assessment with sonography in trauma was not consistently performed and therefore was not included in the analysis. Both groups of patients (received selective imaging versus pan-scan imaging) were compared for hospital length of stay and ICU length of stay. Secondary outcomes included the number of operative interventions, the number of consults based on injury, and the number of consults based on incidental findings, vital signs, and laboratory findings upon presentation to the hospital. This study classifies incidental findings as findings that were not diagnosed during physical exam but were clinically impactful and required action and/or changes in management or treatment during the patient’s hospitalization, as previously defined in prior trauma studies [15].

Statistical analysis

Demographic data and other characteristics of patients are displayed as means and standard deviation (SD) or medians and interquartile range (IQR) for numeric data. Categorical data are expressed as frequencies and percentages. Chi-square or Fisher’s exact test (as applicable) was used to compare the association of categorical variables to one another. Independent sample t-tests or Mann-Whitney U-tests for non-parametric data were used to compare the means between those receiving selective CT versus those receiving a pan-scan on all the contributing factors. Univariate logistic regression was performed to determine an association between each independent variable to the outcome variable, i.e., type of diagnostic testing (selective CT versus pan-scan). Age and sex-adjusted multivariable logistic regression models were then performed using best subsets regression with the criteria of highest R2 and clinically important predictor variables in the model to determine the association between the outcome variables (type of diagnostic testing) and the independent variables while controlling for all the other variables in the model. Simple and multiple linear regressions were performed with hospital length of stay as the outcome variable. Other models were performed to predict CT imaging congruent with complaint and operative intervention. Bonferroni correction was used for multiple comparisons. Beta and 95% confidence intervals (95%CI) and odds ratios (OR) and 95%CI were determined from the univariate and multivariable analyses. Type 1 error rate was set at 5%. SPSS version 29 (IBM Corp., Armonk, NY, USA) was used to perform all analyses. With a threshold of power of 80, we find that for a medium effect size (Cohen’s d = 0.5), the study is well powered for the two primary outcomes after adjustment. Even after adjusting for two primary outcomes with a Bonferroni correction, the power remains high. This confirms that the current sample sizes (SCT = 105, pan-scan = 98) are sufficient for the intended comparisons.

## Results

A total of 509 patients were identified through ICD-10 codes as admitted for GLF from January 1, 2021, to March 30, 2022. Of these, 208 patients were excluded because they had no imaging or were not evaluated by the trauma team. Ninety-eight patients who were evaluated by the trauma team were excluded because they did not meet imaging requirements during their evaluation. The final dataset included 203 total patients (n=105 for selective CT and n=98 for pan-scan) who were eligible to be included in the study. 

The patient characteristics were similar between the selective imaging and pan-scan groups (Table 1). The mean GCS of patients with selective CT was higher, 14.7 (SD=1.0) versus 13.6 (SD=3.2) for patients who had a pan-scan, and which was statistically significantly different (p=0.002). There were no other significant differences in presenting characteristics. 

**Table 1:** Characteristics among patients by type of scan received in the trauma bay (n=203) *P<0.05; Continuous variables are displayed as means and standard deviations (SD) or medians and interquartile ranges (Q3-Q1) for non-normal data. Categorical variables are portrayed as frequency (percent). P-values were obtained by independent sample t-tests or Mann-Whitney U test for non-normal variables (temperature, Glasgow Coma Scale, INR where ⱡⱡ means a significantly higher sum of ranks) and Pearson Chi-Square or Fisher’s Exact Test for categorical variables. BPM=beats per minute; IQR=interquartile range; INR=international normalised ratio

Characteristics	Selective Imaging n=105	Pan-Scan n=98	p-value
Age (years)	82.1 (8.7)	82.3 (9.2)	0.867
Sex			0.298
Female	54 (51.4%)	55 (56.1%)	0.624
Male	51 (48.6%)	43 (43.9%)	0.651
Heart rate (bpm)	80.4 (16.7)	83.4 (20.1)	0.239
Respiratory rate (bpm)	19.0 (3.1)	19.1 (4.2)	0.948
Systolic blood pressure (mmHg)	153.5 (30.0)	150.1 (30.9)	0.467
Diastolic blood pressure (mmHg)	84.9 (15.2)	80.9 (17.6)	0.083
Hemoglobin (g/dL)	12.4 (1.6)	12.0 (2.0)	0.189
INR median (IQR)	1.1 (0.4)	1.1 (0.4)	0.992
Albumin (g/dL)	3.5 (0.4)	3.3 (0.7)	0.061
Glasgow Coma Scale median (IQR)	15.0 (0.0) ⱡⱡ	15.0 (1.0)	0.021*
Injury Severity Score	8.6 (6.6)	9.9 (7.0)	0.164
Anticoagulation	76 (72.4%)	62 (63.3%)	0.089
Dementia	19 (18.1%)	17 (17.3%)	0.623
Intoxicated	6 (5.7%)	5 (5.1%)	0.548

**Figure 1:**
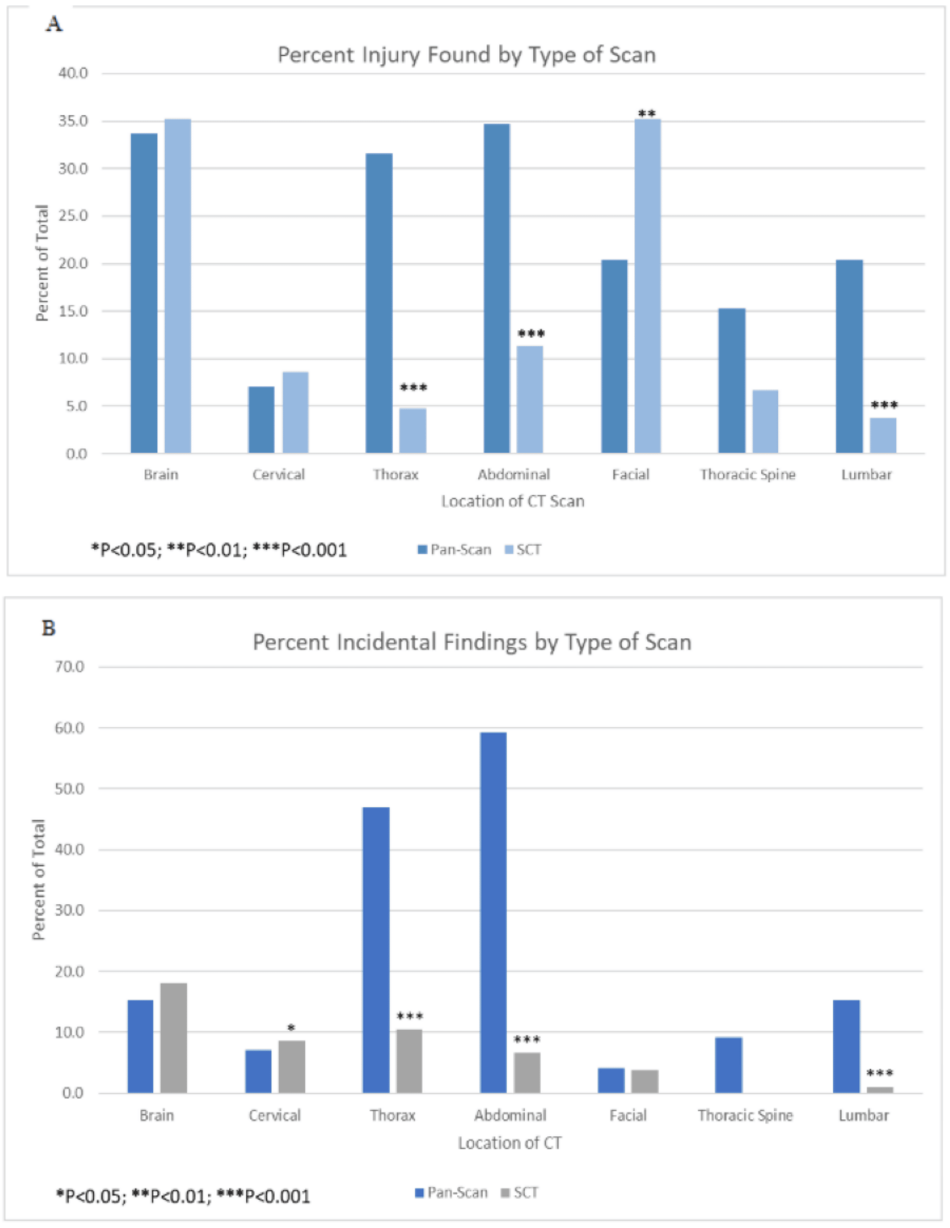
Percent injury and incidental finding by type of scan Percent injury found by type of CT scan (A). Significantly more thoracic spine, abdominal, and lumbar injuries were found with pan-scan, and significantly more facial injuries were found with selective CT (SCT). Percent of incidental findings by type of scan (B). Significantly more thorax, abdominal, and lumbar incidental findings were found on pan-scan, and significantly more cervical incidental findings were found on SCT. There were no differences in injury or incidental findings for the brain from either pan-scan or SCT.

Patient outcomes by type of scan (pan-scan versus selective CT) are displayed in Table 2. Patients receiving a pan-scan had a mean (SD) hospital length of stay of 7.14 days (6.2), significantly longer than selective CT of 4.47 (3.7; p<0.001). Consultations based on injury did not differ significantly between the two groups (p=0.061), but there were significantly more consults based on incidental findings in the pan-scan group, 1.26 (0.9) versus the selective CT group, 0.94 (0.9; p=0.020). Among those receiving a pan-scan, eight (8.2%) had imaging congruent with complaint compared with 83 (79%) of those receiving selective CT (p<0.001, degrees of freedom = 1, effect size = 0.712). Significantly more operative intervention was seen among those who had a pan-scan (n=43, 43.9%) compared with those who had selective CT (n=19, 18.1%; p<0.001, degrees of freedom = 1, effect size = 0.279). Significantly more patients who had a pan-scan went from the emergency department to the ICU (n=40, 40.8%) compared to those who had selective CT (n=29, 27.6%; p=0.003, degrees of freedom = 5, effect size = 0.358). There were significantly more patients from the emergency department to the stepdown unit who had selective CT (n=44, 41.9%) than those who had a pan-scan (n=26, 26.5%; p=0.021, degrees of freedom = 5, effect size = 0.358). Overall discharge disposition was significantly different between pan-scan and selective CT (p=0.013) although there was no significant difference in mortality between those who had a pan-scan (n=4, 4.0%) or selective CT (n=1, 1.0%; p=0.1676, degrees of freedom = 7, effect size = 0.281), but only five patients in total died during the study period. Discharge disposition revealed a significantly smaller proportion of patients who had pan-scan went home when compared to selective CT (n=16 vs 31, 16.3% vs. 29.5%; p=0.026, respectively, degrees of freedom = 7, effect size = 0.281). A significantly higher percentage of injury and incidental findings were found in patients who had a pan-scan of the thorax, abdominal/pelvis, and lumbar (p<0.001 for all in both variables) compared with those who had selective CT (Figure 1A, 1B). A higher percentage of incidental findings (p<0.05) were seen on selective CT for the cervical area compared with pan-scan. 

**Table 2:** Patient outcomes by type of scan (selective CT imaging versus pan-scan) *P<0.05; **P<0.01; ***P<0.001; Continuous variables are displayed as means and standard deviations (SD) or medians and interquartile ranges (Q3-Q1) ⱡ for non-normal data. Categorical variables are portrayed as frequency (percent). P-values were obtained by independent sample t-tests or Mann-Whitney U tests for non-normal variables (hospital length of stay [LOS] and ICU LOS where ⱡⱡ means a significantly higher sum of ranks) and Pearson Chi-Square or Fisher’s Exact Test for categorical variables. LOS=length of stay; IQR=interquartile range

Outcomes	Selective Imaging	Pan-Scan	p-value
Hospital LOS (days) median (IQR)ⱡ	4.0 (3.0)	6.0 (7.0) ⱡⱡ	0.001**
ICU LOS (days) median (IQR)ⱡ	0.0 (2.0)	0.0 (4.0) ⱡⱡ	0.004**
Consults based on injury	0.88 (0.6)	1.08 (0.9)	0.061
Consults based on incidental findings	0.94 (0.9)	1.26 (0.9)	0.020*
Imaging congruent with complaint	83 (79.0%)	8 (8.2%)	<0.001***
Operative intervention	19 (18.1%)	43 (43.9%)	<0.001***
Emergency Department Disposition			<0.001***
Floor	32 (30.5%)	19 (19.4%)	0.069
ICU	29 (27.6%)	40 (40.8%)	0.003**
Stepdown	44 (41 .9%)	26 (26.5%)	0.021*
Home	0	9 (9.2%)	0.002**
Operating room	0	3 (3.1%)	0.069
Other	0	1 (1.0%)	0.303
Discharge Disposition			0.013*
Home	31 (29.5%)	16 (16.3%)	0.026*
Expired	1 (1.0%)	4 (4.0%)	0.168
Home Health	18 (17.1%)	12 (12.2%)	0.327
Skilled Nursing Facility	28 (26.7%)	31 (31.6%)	0.441
Hospice	9 (8.6%)	11 (11.2%)	0.535
Rehabilitation facility	14 (13.3%)	15 (15.3%)	0.682
Against medical advice	4 (3.8%)	1 (1.0%)	0.197
Other	0.0 (0.0%)	8 (8.2%)	0.003**

Regression models 

Univariate and multivariable regression models predicting hospital length of stay are portrayed in Table 3. In the multivariable model, having a pan-scan increased hospital length of stay (β=1.49 [95%CI=0.06, 2.91]; p=0.041) compared with having selective CT. ICU length of stay also significantly increased hospital length of stay (β=0.82 [0.70, 0.95]; p<0.001). A lower hemoglobin upon admission increased hospital length of stay (β=-0.38 [-0.67, -0.08]; p=0.014). Having more consults based on incidental findings increased hospital length of stay (β=0.55 [0.03, 1.07]; p=0.037) but more consults based on injury did not significantly increase hospital length of stay (p=0.800). Other covariates in the model that were not significant contributors to hospital length of stay were CT congruent with complaint (p=0.543), GCS (p=0.787), sex (p=0.737), and age (p=0.489). The full regression model had an R2 of 0.603, meaning 60.3% of the variability in hospital length of stay was accounted for by the factors included in the model. 

**Table 3:** Univariate and multivariable linear regression modeling the outcome of hospital length of stay (LOS) with type of scan as the main predictor variable B= beta coefficient; Consult BOI=Based on injury; Consult BOIF=Based on incidental findings; CT congruent=CT congruent with complaint; Hgb admit=hemoglobin on admission; CT=computed tomography; GCS=Glasgow Coma Scale *P<0.05, **P<0.01, ***P<0.001

Factors	Univariate		Multivariable N=185	
	B (95% CI)	P-value	B (95% CI)	p-value
Type of scan				
Selective CT (Ref)	1		1	
Pan Scan	2.67 (1.24, 4.10)	<0.001***	1.49 (0.06, 2.91)	0.041*
ICU LOS	0.88 (0.76, 0.99)	<0.001***	0.82 (0.70, 0.95)	<0.001**
Sex	-0.06 (-1.54, 1.42)	0.936	-1.78 (-1.22, 0.87)	0.737
Age	0.03 (-0.58, 0.11)	0.540	-0.02 (-0.08, 0.04)	0.489
GCS	-0.45 (-0.75, -0.16)	0.002**	0.03 (-0.18, 0.24)	0.787
Hgb admit	-0.56 (-0.97, -0.16)	0.007**	-0.38 (-0.67, -0.08)	0.014*
Consult BOI	1.14 (0.14, 2.14)	0.026*	0.09 (-0.60, 0.77)	0.800
Consult BOIF	1.55 (0.83, 2.27)	<0.001***	0.55 (0.03, 1.07)	0.037*
CT congruent	-2.05 (-3.50, -0.60)	0.006**	0.43 (-0.97, 1.83)	0.543
				R2=0.603

Univariate and multivariable models to predict having a pan-scan are shown in Table 4. In the multivariable model, a longer hospital length of stay significantly predicted pan-scan with an OR of 1.15 [1.03, 1.28]; p=0.017. This means that the longer the hospital length of stay, the more likely the patient had a pan-scan. GCS was a significant factor with an OR of 0.77 [0.61, 0.95; p=0.013]. This means that the lower the GCS, the more likely a patient had a pan-scan. Consultations based on injury were also a significant factor with an OR of 1.69 [1.03, 2.74; p=0.036]. This means that for each consult given based on the patient’s injury, the patient had a 69% higher chance of having had a pan-scan. Multivariable models were performed with CT imaging congruent with patient complaint (Appendix 1) and operative intervention (Appendix 2) as the outcome variables. Controlling for hospital length of stay, operative intervention, sex, and age, patients who had a pan-scan compared to those having selective CT were less likely to have the CT image congruent with their complaint (OR 0.24 [0.10, 0.62; p<0.001]). Having a pan-scan made patients 3.53 times as likely (OR 3.53 [1.85, 6.72; p<0.001]) to undergo operative intervention versus those who had selective CT when adjusting for age and sex. 

**Table 4:** Univariate and multivariable logistic regression models depicting which factors predict having a pan-scan. *P<0.05; **P<0.01; ***P<0.001; Multivariable model was adjusted for all variables in the table. LOS=length of stay; ICU=intensive care unit; GCS=Glasgow Coma Scale; Consult BOI=Consult based on injury; Consult BOIF=Consult based on incidental findings; Hgb admit=hemoglobin on admission. The more consults based on injury, the more likely to have had a pan-scan. The higher the GCS, the less likely to have had a pan-scan, and the longer the hospital length of stay, the more likely to have had a pan-scan when everything is controlled for in the model.

	Univariate			Multivariable		
Factors	OR (95% CI)	Wald	P-value	OR (95% CI)	Wald	p-value
Hospital LOS	1.14 (1.05, 1.24)	10.56	0.001**	1.15 (1.03, 1.28)	5.74	0.017*
Sex	1.21 (0.70, 2.10)	0.45	0.503	1.20 (0.61, 2.35)	0.29	0.593
Age	1.00 (0.97, 1.03)	0.03	0.867	1.00 (0.96, 1.03)	0.05	0.817
ICU LOS	1.12 (1.01, 1.23)	4.94	0.026*	0.95 (0.84, 1.09)	0.51	0.477
GCS	0.78 (0.65, 0.94)	7.00	0.008**	0.77 (0.61, 0.95)	6.14	0.013*
Consult BOI	1.44 (0.98, 2.13)	3.42	0.064	1.68 (1.03, 2.74)	4.38	0.036*
Consult BOIF	1.44 (1.05, 1.97)	5.07	0.024*	1.24 (0.88, 1.76)	1.48	0.224
Hgb admit	0.90 (0.77, 1.05)	1.72	0.190	1.01 (0.83, 1.23)	0.01	0.930

## Discussion

Deciding whether to have an older adult patient undergo a pan-scan or selective CT after blunt trauma remains a challenging position for trauma surgeons with conflicting evidence in the literature [6-8]. CT plays a critical role in diagnosing injuries in patients who present with high-energy impact and no physical examination abnormalities. It is often difficult to decide what type of scan to perform in these patients, which brings forth an important discussion in older adults presenting with low-energy trauma as these patients are being admitted to the hospital for GLF more frequently [16-18] and experience increased mortality [17]. There have been recent studies that have examined similar topics. One particular study showed that physical exam was not an accurate predictor of injury in patients >65 years old [19]. This study differs in the inclusion of patients who did not undergo pan-scan as well as their outcomes. There was also another study that contradicted the results of the aforementioned study and supports the judicious use of CT scans in low-energy mechanisms [20], but that study focused more on the use of physical exam as a guide to selective imaging, whereas this study focuses primarily on the outcomes secondary to selective imaging. 

This study evaluated the direct comparison between pan-scan imaging in older patients after a GLF and selective CT imaging for all relevant measures to determine varying outcomes in both groups while controlling for applicable and confounding covariates. Our study highlighted some of the negative consequences of pan scanning, specifically within the elderly population, including longer hospital and ICU length of stay, and more incidental findings compared with having selective CT imaging. Other investigators found similar hospital length of stay and ICU length of stay for patients receiving pan-scan or selective CT, contradicting our findings on these factors [21], but again, those investigators included patients of all ages while we only studied older adults. 

The increased length of stay could in part be due to increased incidental findings. Niedermeier et al. also found higher incidental findings (73.9%) in older patients with low-energy falls receiving emergent CT scans [9,22] with no difference in mortality compared to patients who received selective CT [6,7,11,12]. Additionally, having a pan-scan increased the number of consults, and our multivariate analysis showed that the number of consultants was an independent predictor of hospital length of stay. 

When looking at the operative interventions, we found that not all operative interventions performed on patients who received a pan-scan were related to injuries. Fourteen percent of patients in the pan-scan group had procedures and further testing unrelated to original injuries including implantation of a dual chamber pacemaker, percutaneous catheterization of the left common femoral artery, placement of a catheter into the aorta, diagnostic abdominal aortography placement of catheter of the right external iliac artery and selective angiography of the right iliofemoral arteries, open repair of the right femoral artery pseudoaneurysm, cystoscopy with the placement of a Foley catheter, two colonoscopies (colon mass findings), incidental colon mass and procedure, cardiac ablation for atrial fibrillation, esophagogastroduodenoscopy, incidental rectal malignancy, interventional radiology-guided biopsy related to an incidentally found lung mass, and pericardiocentesis related to cardiac effusion. None of the patients in the selective CT group had other procedures or testing not related to their injuries. 

While the incidental findings discovered on pan-scans do not always carry clinical significance [7,9,10], physicians may prefer their utilization to improve confidence in the diagnoses. Harntaweesup et al. found that the confidence in the diagnosis of a high-energy blunt trauma was 100% for all physicians (n=22) among patients who had a pan-scan compared to 13.6% confidence (n=3) before the pan-scan [23]. Our patient population is considerably different as we studied older adults with low-energy blunt trauma from a GLF, making the clinical assessment for diagnosing and treating these patients more specific. Overall, this study calls into question the necessity of routine use of pan-scan imaging on older patients presenting with GLF, but ultimately it is the opinion of this group that the decision should be left to the judgment of the physician. This study also reinforces the importance of a thorough evaluation and tertiary survey, as the extra CT in selective imaging is dependent on the patient’s symptoms and notable exam findings. 

There were some limitations to this study. This was a retrospective study and therefore cause and effect cannot be determined temporally. For example, documentation in the medical records was not always clear if the patient arrived with the complaint and then was scanned or had a scan and then asked about the injury found. While the paper did look at mortality between both groups, the total mortality would have been underpowered as a primary outcome in this study. Future studies could evaluate mortality from low-energy mechanism between the two groups across multiple centers. Future studies could also evaluate possible cost analysis between the two groups, given an insignificant difference in mortality. This study was performed at one institution, making the results not necessarily generalizable to the population at large. However, the strength of this study was the robust analysis using regression models to determine the association of pan-scans for GLF of older adult patients with numerous relevant clinical factors compared with selective CT. We also modeled hospital length of stay, operative intervention, and CT imaging congruent with patient complaint when using pan-scan versus selective CT as the main predictor variable in each model and were able to show significant differences in pan-scan compared with selective CT. Another strength was the lack of discrimination between different ground-level falls. 

## Conclusions

In conclusion, patients who had selective imaging had shorter hospital and ICU length of stay, required fewer operative interventions and consults based on incidental findings or injury but had more imaging congruent with complaint but no significant difference in mortality compared to patients who had a pan-scan. While using selective scans does result in the outcomes listed above, there is always a risk of missing injuries. Using a clinical picture, such as thorough history and physical, as a guide for diagnostic imaging in patients after ground-level falls reduces the number of unnecessary imaging without compromising patient outcomes and may reduce hospital length of stay.

## Appendices

Appendix 1 

**Table 5:** Predicting CT imaging congruent with patient complaint *P<0.05; **P<0.01; ***P<0.001; HLOS=hospital length of stay; Op=operative

Factors	OR (95% CI)	Wald	p-value
Pan-Scan vs. Selective CT	0.24 (0.10, 0.62)	60.67	<0.001***
HLOS	0.99 (0.91, 1.09)	0.01	0.935
Op intervention (Yes vs. No)	0.71 (0.28, 1.81)	0.53	0.467
Sex (Female vs. Male)	0.69 (0.30, 1.62)	0.72	0.397
Age	0.99 (0.94, 1.04)	0.30	0.583

Appendix 2

**Table 6:** Predicting operative intervention with pan-scan versus selective CT *P<0.05; **P<0.01; ***P<0.001

Factors	OR (95% CI)	Wald	p-value
Pan-Scan vs. Selective CT	3.53 (1.85, 6.72)	14.66	<0.001
Age	0.97 (0.93, 1.01)	2.77	0.096
Sex (Female vs. Male)	1.66 (0.85, 3.25)	2.19	0.139
